# Study protocol for a randomized trial examining a peer grief support approach for people grieving drug overdose deaths

**DOI:** 10.21203/rs.3.rs-6959900/v1

**Published:** 2025-08-21

**Authors:** Karen Chan Osilla, Katherine E. Watkins, Tanya Lord, Glen Lord, Franklin Cook, Katherine Nameth, Jane P. Kim, Wendy Hawkins, Alison Athey

**Affiliations:** Stanford University School of Medicine; RAND; Peer Support Community Partners; Peer Support Community Partners; Peer Support Community Partners; Stanford University School of Medicine; Stanford University School of Medicine; RAND; RAND

**Keywords:** Drug overdose, death, peer support, grief, family therapy

## Abstract

**Background:**

People bereaved by drug overdose experience a “special grief” that includes guilt, shame, and blame for their loved one’s death, which often compounds suffering and interferes with help-seeking. This population is vulnerable to poor health outcomes including prolonged grief disorder, risky substance use, and suicidal thoughts and behaviors. Peer grief support may reduce risks in people bereaved by overdose by buffering grievers from the negative effects of increased stigma and isolation. Peer Support Community Partners has developed the RIVER peer grief support model for bereavement. The model has been implemented successfully in the community and for drug overdose bereavement, but has not yet been rigorously tested.

**Methods:**

The current study is a two-phased project that aims to test whether the RIVER peer grief support approach improves health outcomes in those grieving drug overdose deaths. In the first phase, we will prepare for and conduct a pilot test of the RIVER peer grief support approach. We will also assess an innovative mechanism for engaging grievers through Medical Examiners Offices (MEOs). In the second phase, we will compare enhanced usual care (EUC) to EUC plus RIVER peer grief support (RIVER) in a randomized controlled trial.

**Conclusions:**

The study aims to build the science of overdose bereavement and provide increased support for a vulnerable population of grievers.

**Trial registration:**

https//clinicaltrials.gov/study/NCT06854757

## 1. Introduction

The United States (US) is in an overdose crisis. An estimated 110,000 Americans died by drug overdose in 2023(1), putting the national total since 2000 at more than 1.1 million overdose deaths (2). Overdose death rates have accelerated as the crisis wears on and as more deadly drugs such as fentanyl make their way into the drug supply (3). The overdose crisis has had profound, negative effects that extend beyond the tragedy of the deaths themselves. For example, the overdose crisis has increased the burden on health, foster care, and criminal-legal systems (4) and spurred on syndemics (5) of suicide (6), infectious disease (7), and violence (8, 9). Research has focused on the broad effects of the overdose crisis on society but has neglected the impact on people who are left behind by drug overdose deaths and the potential role that overdose bereavement may play in fueling risky substance use among the bereaved (10).

Recent findings highlight it is common for people to lose someone to overdose. About 42% of US (i.e., more than 125 million people) personally know someone who died by overdose (11). More than 32% of adults report that they have lost a loved one to an opioid overdose (12). Children have also been heavily impacted by overdose loss. In 2019, more than 1.4 million US children lost a family member to overdose, including 317,000 who lost a parent (13). To put these data into perspective, the prevalence of exposure to overdose deaths is estimated to be more than three times higher than the prevalence of exposure to suicide (14) and is on par with rates of bereavement by COVID-19 deaths (15). Without intervention, the ripple effects of overdose loss will persist, affecting millions of individuals and communities.

People who are bereaved by overdose are vulnerable to poor mental health outcomes. They experience high rates of prolonged grief disorder (PGD) (16, 17), a type of grief that lasts longer and has more severe effects than other types of grief and confers increased health risks (18). Rates of PGD are higher among those bereaved by overdose compared to those who have lost someone to suicide, homicide, and other unintentional injuries (19). Overdose bereavement may also increase risk for other mental health problems. Cross-sectional studies suggest that overdose bereavement is associated with the development of posttraumatic stress disorder, depression, anxiety, physical pain (20), as well as suicidal thoughts and behaviors (17, 21–23).

Overdose bereavement may also increase risk for substance misuse and overdose, especially in those who struggle to heal after an overdose loss. People who experience grief, particularly prolonged grief, are at increased risk for developing their own substance use problems (24–26), perhaps because they struggle to emotionally process and accept the loss (27) or because they use substances to cope. People who use drugs (PWUD) before their loss may be particularly at risk for poor health outcomes because substance misuse itself appears to increase the risk for problems with grief (24, 25). One study showed that while overdose bereavement led some PWUD to engage with harm reduction practices, most continued to use substances in risky ways (28). Substance use may fuel substance-related morbidity and mortality among the bereaved by leading some to initiate substance misuse and others to experience increases in the severity of their substance use disorders.

Although no research has evaluated the association between overdose bereavement and a subsequent overdose among the bereaved, a study of Norwegian parents who lost children to overdose revealed these grievers were at more than double the risk for external injury deaths (i.e., accidents, suicides, or homicides) (29). Studies focused on bereaved PWUD show that this subset of grievers are also at increased risk for their own experiences of non-fatal or fatal overdose (27, 30). Overdose bereavement may perpetuate the overdose crisis by increasing risk for overdose morbidity and mortality in people left behind.

The traumatic nature of overdose loss and stigma associated with overdose may make healing from grief more difficult (16)^32^). The Dual Process Model of grief suggests that healing can occur when grievers balance both processing the loss and restoring their lives after loss (21, 32). People bereaved by overdose may struggle to emotionally process and accept the loss due to intense, negative emotions, including shame and guilt (10, 16, 33–35). Some may also struggle to accept the loss if there is ambiguity surrounding the circumstances of the death or confusion about whether the overdose was suicidal or homicidal in nature (21, 36). Stigma may also interfere with the emotional processing of the loss by exacerbating guilt and shame (37). Research comparing overdose bereavement to other traumatic losses shows that guilt and shame are significantly more severe among people bereaved by overdose than among people bereaved in other ways (e.g., a cancer-related death) (10, 16, 34, 38). Stigma may be especially common and harmful to people with personal and family histories of drug use (28, 34). The experience of stigma and intense emotions interfere with meaning making and healing after loss (39) and may contribute to mental health conditions such as depression (17, 33). Addressing the intense negative emotions that inhibit acceptance of the loss and the experiences of stigmatization may help people bereaved by overdose heal.

People bereaved by overdose are in critical need of resources as they often lack support for restoring their lives after loss (40). Informal social support from friends and family appears to be a critical component of healthy grieving by promoting the restoration of the lives of bereaved people (40). Unfortunately, perceived stigma interferes with healing by promoting social withdrawal and negatively impacting the relationships of people bereaved by overdose (34, 41–43). People bereaved by overdose, especially bereaved PWUD, also face barriers to engaging with professional supports (27). While most people bereaved by overdose report that they would benefit from professional help, only one-third report they have received professional support (44). In the absence of informal and professional support, it can be difficult for those bereaved by overdose deaths to effectively cope with the loss.

Peer grief support interventions may be protective against PGD, substance use, and mental health outcomes. Peer interventions involve “giving and receiving help founded on key principles of respect, shared responsibility, and mutual agreement” (45). A review found that peer support was associated with reductions in risk for PGD and with improved well-being in people bereaved by sudden or violent deaths (46, 47). Importantly, people who receive peer grief interventions experience reductions in substance use compared to grievers who do not receive peer support (48–50). Qualitative studies of this population suggest that peer interventions may serve as an antidote to the trauma and stigma of overdose bereavement by promoting connectedness, acceptance of the loss, and adaptive coping (42, 51). Research also demonstrates that peer-facilitated interventions can improve outcomes for the facilitators themselves evidenced by greater posttraumatic growth, increased social connectedness and reduced adverse grief outcomes in those facilitating a peer grief intervention (52, 53). However, the evidence supporting peer grief support interventions for overdose bereavement is scarce. To our knowledge, no peer reviewed research has evaluated the effectiveness of peer interventions for preventing PGD and substance misuse in people who are bereaved by overdose.

**Figure 1:**
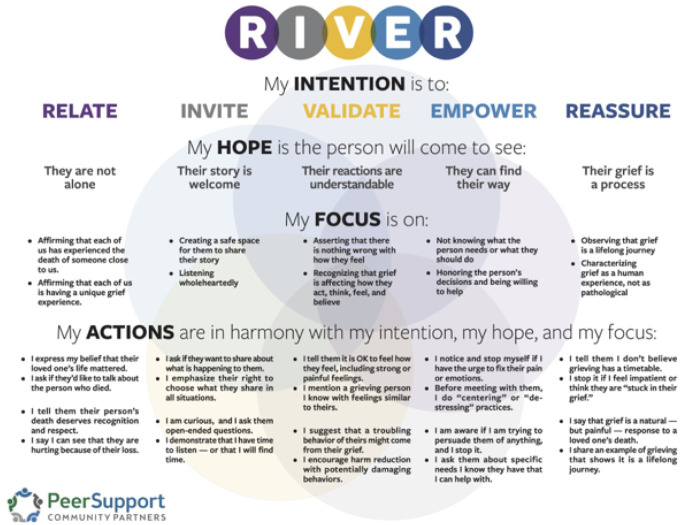
RIVER peer grief support model

To address this gap, researchers from academic institutions have collaborated with a peer grief support organization [i.e., Peer Support Community Partners (PSCP)] in the current study to advance the science of overdose bereavement. PSCP has developed and implemented a peer grief support model called RIVER (relate, invite, validate, empower, reassure) for people bereaved by overdose. The RIVER model (Fig. 1) was developed by people who experienced traumatic bereavement, including bereavement by overdose, after decades of learning from and delivering peer grief support. The RIVER model distills this learning, evidence-based peer support practices, and trauma-informed approaches into a process intervention. Rather than prescribing specific intervention activities, the RIVER model of peer grief support guides peer grief allies, or supporters in *how* to engage with people bereaved by overdose. Peer grief allies are trained to use their lived experience to *relate* to people who are bereaved by overdose, *invite* them to make meaning of the loss by sharing their grief story and demonstrating that their story is welcome, *validate* their grief to show that their reactions are understandable, *empower* grievers to engage in restorative coping so that they can restore their lives after loss, and *reassure* them grief is a lifelong journey and process. While the RIVER peer grief support model was developed without specific attention to the Dual Process Model of coping with grief, RIVER closely aligns with the model because RIVER promotes balancing both loss- and restoration- related bereavement needs. This study will be the first to test the impact of the RIVER peer grief model on grief, substance use, and mental health outcomes of people bereaved by overdose.

To address evidence that most grievers lack access to grief support, this study established partnerships with Medical Examiners’ Offices (MEOs). MEOs are legally mandated to conduct death investigations following every overdose death and to make death notifications to the legal next-of-kin to those who die by overdose. As part of the death investigation and notification process, MEOs routinely interact with the next-of-kin to overdose decedents. Indeed, MEOs may be the only organizations that systematically compile the contact information of all next-of-kin to overdose decedents within their jurisdictions. Partnering with MEOs may provide a scalable path to proactively engage people bereaved by overdose before they develop poor grief-related outcomes. This study will be the first to evaluate the feasibility, acceptability, and appropriateness of proactively engaging people bereaved by overdose via MEOs.

### 1.1 Study Aims

The current study has three aims. In aim one, we will adapt training materials for the RIVER peer grief support model to fit the needs of the communities surrounding our partnering MEOs, develop a fidelity measure, and create workflows for engaging grievers and connecting them with peer grief allies. In aim two, we will conduct a pilot test to evaluate the acceptability and feasibility of engaging grievers via MEOs, refine the fidelity measure, and evaluate if peer grief support using the RIVER model was satisfactory to grievers. In aim three, we will conduct a randomized controlled trial (RCT) to evaluate the effectiveness of the RIVER peer grief approach plus enhanced usual care (RIVER + EUC) to prevent PGD and substance misuse in people bereaved by overdose, over and above any effects of EUC. Participants will be recruited from MEOs and will complete baseline, 3, 6, and 12-month surveys on (a) proximal outcomes (i.e., connectedness, acceptance, and coping) and (b) long-term outcomes (i.e., PGD and substance misuse). Post-RCT qualitative interviews of study participants and mixed methods assessments with peer grief allies will supplement data collection and allow us to understand the characteristics of the RIVER peer grief approach that drive effectiveness and its impact on peer grief allies.

## 2. Methods

### 2.1. Overview of study procedures

**Figure 2:**
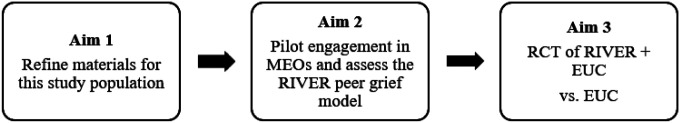
Study aims

First, we will supplement the already-developed RIVER peer grief approach with materials needed to rigorously test its effectiveness (Aim 1; see Fig. 2). We will engage three advisory boards (family, research, and medical examiners) to (1) develop recruitment materials and procedures for recruiting grievers who interact with MEOs, (2) adapt RIVER training materials to local MEO regions, and (3) develop fidelity measures. Next, we will conduct a pilot test of the RIVER peer grief support model with at least twenty people who are bereaved by a drug overdose death and recruited through MEOs. We will determine the feasibility and acceptability of griever engagement procedures and materials from the perspective of grievers and MEOs, as well as grievers’ satisfaction with peer grief support that uses the RIVER model (Aim 2).

In our third aim, we will use an RCT to test the effects of RIVER plus EUC compared to EUC alone for people who are bereaved by a drug overdose death and recruited from MEOs (Aim 3). People bereaved by a drug overdose death will be randomized to receive RIVER plus EUC or the EUC alone condition. RCT participants (*N* = 320 at baseline; 208 at 12-month follow-up) will be assessed on their experience of proximal and distal grief, substance use, and mental health outcomes at baseline, and during 3-, 6-, and 12-month-follow-ups. We will also conduct qualitative interviews with a subset of participants at the end of the study to better contextualize the mechanisms in which the RIVER model affected them. Finally, we will collect qualitative and quantitative data from peer grief allies at the beginning and end of the RCT to explore how delivering peer grief support affects their well-being.

### 2.2. RIVER peer grief model

Grief work using the RIVER peer grief support model is dynamic and varies based on the individual’s specific circumstances, such as their relationship to the decedent (e.g., whether the relationship was strained) and the circumstances around the death (e.g., whether the death was anticipated), as these factors can substantially impact grief and substance use outcomes in the bereaved (21). At minimum, a peer grief ally will meet with a griever for at least three 45- to 75-minute virtual sessions to deliver the RIVER peer grief support. Meetings range in frequency from weekly to monthly (often less over time), and it is common for the meetings to continue for at least a year. These ranges are variable, depending on the wishes and agreement of the griever and the peer grief ally. Peer grief allies will also provide grievers with the same materials that are part of the EUC, which include a psychoeducational booklet about grief (54) and a local mental health and grief support resource list.

#### 2.2.1 Peer grief ally training and supervision

PSCP has a process for onboarding peer grief allies that assesses their readiness to deliver peer grief support given their own bereavement, their willingness to engage in ongoing supervision, their completion of at least 16 hours of training, and the results of background checks. The 16-hour training is broken into two parts. The first half of training focuses on using the RIVER peer grief support approach. Peers are trained in this process model through a combination of didactic information, discussion, and role plays. Peers are trained to identify clinical problems (e.g., posttraumatic stress disorder) and risks (e.g., dangerous substance use, suicidal crises) and taught how to connect people to clinical and emergency services as needed. The second part of the training addresses ways that delivering the RIVER peer grief support model could affect peers’ own well-being and grief process, both negatively through re-traumatization and positively through posttraumatic growth. Peers are informed about well-being resources available through PSCP and are encouraged to use these if they experience re-traumatization or other negative impacts of peer grief work.

RIVER supervisors meet individually with peers each week to coach them in their delivery of the RIVER peer grief support model and review documentation. As part of this study, supervisors will review and discuss audio recordings of sessions. Supervisors will also explore how grief support delivery impacts the peers’ well-being (e.g., identifying re-traumatization, risky substance use, etc.). Peers are advised to engage with a licensed clinician at PSCP if they experience re-traumatization and to attend monthly group reflective supervision in which they reflect with other peer grief allies on how their work affects their own grief journeys.

#### 2.2.2. Enhanced Usual Care (EUC) for Grief Support

The EUC condition involves proactive outreach to supply grievers with existing, published psychoeducational booklet about grief and a list of grief support resources in their community. We will provide participants a psychoeducational booklet about grief developed by an organization called *What’s Your Grief* (54). This 12- page booklet was developed for a lay audience of people bereaved by any sort of death (i.e., not specifically a drug-related death) and has been used by PSCP in their general and overdose specific bereavement work (55). We will distribute booklets in either English or Spanish, depending on the preference of the griever. We will also provide participants with a list of local grief, mental health, and substance use treatment resources.

### 2.3. Setting

We will primarily conduct our study in regions that have the highest prevalence of overdose bereavement [i.e., New England and the central Southeast (11)]. We will also conduct the study in the Pacific region, where the prevalence of overdose bereavement is lower compared to other US regions, to account for the possibility that grievers experience stigmatization, isolation, and a dearth of grief resources in communities where overdose bereavement is less common. We will partner with MEOs in the state of Connecticut; Jefferson County, AL; and San Diego County, CA. Despite differences in the prevalence of overdose bereavement in these communities, the number of overdose deaths in each region is high. Partnering with MEOs in these locations allows us to bolster bereavement support services in communities deeply impacted by drug overdose deaths.

### 2.4. Aim 1 Procedures

We will take a process-oriented (56) approach to optimizing the RIVER peer grief support model that PSCP has already created, while maximizing both internal and external validity as we prepare for rigorously evaluating the RIVER model’s effectiveness. First, we will refine RIVER training materials. We will work with local MEOs to customize training materials as informed by the local context (e.g., encouraging cultural humility, sharing local trends in next-of-kin demographics, describing how drug use has affected their local community, how to address grievers who use substances themselves). Second, we will develop measures of fidelity and adherence to the RIVER peer grief support model. Like motivational interviewing (57), RIVER is a process model that describes the style in which an intervention is given. Thus, we will develop a RIVER fidelity assessment and coding manual similar to the Motivational Interviewing Treatment Integrity (MITI) measure (58) whereby coders rate peer grief facilitator speech on each of the RIVER domains (relate, invite, validate, empower, reassure) using a 5-point Likert scale. Finally, we will work with our MEO partners to develop recruitment workflows and establish data sharing plans to support proactive outreach to people bereaved by overdose by our research data collection staff.

### 2.5. Aim 2 Procedures

**Figure 3:**
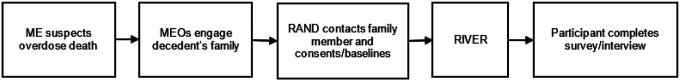
Study design

We will work with our three partnering MEOs to recruit 20 people bereaved by overdose for a pilot study of the RIVER peer grief support model (see Fig. 3). We will recruit one bereaved adult per decedent to participate in study assessments but, in keeping with PSCP’s inclusive practice, we will deliver the RIVER peer grief support model to other grievers who lost the same decedent upon request. Participants will be people who (1) lost a loved one to an unintentional drug overdose death within the past year, (2) are age 18 and older, (3) fluently understand English or Spanish, and (4) have the capacity to give consent (e.g., excluding those with severe cognitive impairment, those in active psychosis, and those with developmental disabilities). Based on early feedback from our Medical Examiners Advisory Board, we will operationalize drug overdose deaths as those caused by intoxication from illicit substances (e.g., heroin), prescription substance misuse (e.g., using opioids in a way that was not prescribed), and/or counterfeit pills. Deaths in which intoxication exacerbated underlying medical conditions will be considered drug overdose deaths. Deaths that result from an adverse reaction to prescription medications that were used as prescribed (e.g., allergic reaction) will not be considered drug overdose deaths.

The study will exclude people who (1) need hospitalization for psychiatric symptoms or substance use disorders, (2) have active suicidal ideation, (3) lost someone to a drug overdose death more than one year ago, or (4) lost someone to a drug overdose death that was suicidal in nature or of undetermined intent. Those who use substances but do not require hospitalization for substance use disorder will *not* be excluded given the connection between substance use and poor grief outcomes. We will include only those grieving death that occurred within the past year because prolonged grief disorder can be diagnosed no earlier than 12-months after a loss (59) and we aim to evaluate the effectiveness of the RIVER peer grief support model in preventing this outcome. On the recommendation of our advisory boards, we will exclude people bereaved by suicide and deaths of undetermined intent because the bereavement experiences of these populations differ from those bereaved by unintentional drug overdose deaths.

We will proactively recruit people bereaved by overdose from MEOs. MEOs routinely identify and contact the legal next-of-kin to overdose decedents in order to provide them with death notifications. At the participating MEOs, nearly all manner and cause of death rulings are complete within 60 days of receiving the decedents’ remains. After this determination, staff at our partnering MEOs will ask families bereaved by overdose to give consent to be contacted by the study team. MEO or study staff will provide grievers with a postcard that describes the study, provides a QR code that links grievers with a page with additional study details, and includes contact information for the study team. Study engagement procedures will be carried out by MEO staff including medical examiners, death investigators, and chaplains.

Next, the study team will engage potentially eligible grievers. We will contact grievers to describe the study, screen them for eligibility, conduct the informed consent process, gather baseline data, and send the psychoeducational materials and list of local grief resources that make up EUC. The study team will wait until at least 90 days after families receive the death notification to contact grievers. This delay in outreach was recommended by PSCP and our advisory boards who suggested that grievers may be focused on making funeral arrangements and addressing immediate stabilization needs in the immediate aftermath of loss. However, grievers who contact the study team earlier may be enrolled earlier upon their request.

Enrolled participants will be referred to PSCP for peer grief support. PSCP will review grievers’ information and match them with a peer grief ally. PSCP will contact the participant within three business days to schedule the first RIVER peer grief support session. During the first session, RIVER peer grief allies will schedule at least three regular sessions over the initial three months after enrollment at a cadence agreed-upon the peers and participants. All sessions will be conducted virtually or by phone. Sessions will be audio recorded to support peer grief ally supervision and fidelity assessment.

Three months after baseline, the study team will conduct 45-minute interviews with the participant. Participants will complete the Feasibility Intervention Measure (FIM) (60) that tests whether an intervention can be successfully used or carried out within a given setting. The FIM includes questions about how workable, possible, doable, and easy the RIVER peer grief support model was for the participant. Acceptability will be evaluated using the Acceptability of Intervention Measure (60) that tests how agreeable, palatable, or satisfactory the intervention was. Finally, we will use the Client Satisfaction Questionnaire (CSQ) (61, 62) where scores of at least 26 of 32 (81%) indicate acceptability of an intervention (63). Questions include whether the intervention was a quality service, met their needs, and helped them cope. Participants will be asked open-ended questions about their experience with recruitment (e.g., “What did you like or not like about that process?”) and peer grief support (e.g., “What did you think of your discussions with your peer grief ally? How can we make the discussions helpful to people from different backgrounds?”). Interviews will be recorded and transcribed. Participants will be compensated $50 at baseline and $75 at the follow-up interview.

#### 2.5.1. Aim 2 Analyses

We will quantitatively analyze engagement, feasibility, acceptability, and satisfaction using summary statistics such as rates or means, as appropriate. Qualitative interviews will be coded by two coders to identify common themes using content analysis in Dedoose. We will also conduct a rapid content analysis (64) to efficiently glean insights that can be incorporated while still recruiting for the study. These methods include having a first coder writing detailed notes during each interview and categorizing across comments about the feasibility and acceptability of recruitment as well as the RIVER peer grief support model. Then, a second coder validates the codes after listening to the interview recording. Analysts will meet regularly to discuss any differences and align on ratings. Coding will be iterative and will include comments that may be positive, negative, or neutral. Classic content analysis will be used to categorize quotes within each concept into themes (e.g., proactive outreach from the MEO was helpful) (65). We chose a sample size of 20, however sample size will ultimately be determined by thematic saturation in qualitative analyses, at which point data collection becomes redundant (66). If needed to form consensus themes, we will recruit additional participants for the pilot. We will follow procedures to maintain rigor of qualitative analyses, including an audit trail and data triangulation following the Standards for Reporting Qualitative Research (2014) (67) for data collection and analysis.

### 2.6. Aim 3 Procedures

We will recruit people who are bereaved by overdose from our three partnering MEOs for an RCT comparing the RIVER peer grief model plus EUC to EUC alone. Participants will complete assessments at baseline and 3-, 6-, and 12-month follow up. The inclusion and exclusion criteria for the RCT will be identical to Aim 2 unless advised otherwise by the advisory boards. We aim to recruit 320 people with a goal of 65% retention (208 people) at the 12-month follow-up.

We will also recruit ten RIVER peer grief allies to complete a baseline and follow-up survey prior to and after the RCT. They will also be invited for a brief qualitative interview post-RCT to assess their experiences delivering peer grief support.

#### 2.6.1. Randomization and Stratification

Upon completion of the baseline survey, participants will be randomly assigned to the RIVER peer grief support model plus EUC or to EUC alone. Randomization will involve a stratified permuted block randomization with random size blocks. This ensures the number of people allocated to each group is approximately equal throughout recruitment (68). We will stratify the sample by history of opioid misuse (lifetime heroin use and/or lifetime prescription opioid misuse) (69), under the premise that (1) drug overdose bereavement among PWUD may increase risk for future drug overdose deaths which typically involve opioids and (2) PWUD may experience grief problems that are more severe or different from people bereaved by a drug overdose death who do not use drugs. Randomization will be performed within each stratum.

#### 2.6.3. Measures

**Figure 4:**
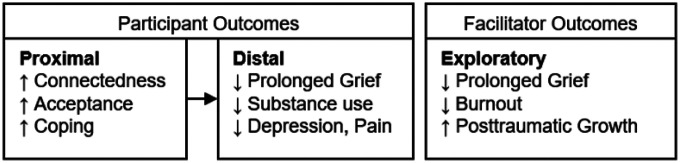
Hypothesized outcomes

All measures will be collected using surveys administered by an interviewer over the phone. We have selected measures that are reliable and valid in populations experiencing grief, as well as measures from the NIH HEAL Initiative Common Data Elements Repository and the NIH PhenX Toolkit (70, 71) (see Table 1). Our primary outcome is PGD, and the associated outcomes that we are evaluating have previously demonstrated a relationship with the severity of grief symptoms experienced. We also have additional proximal and distal outcomes that will serve as pre-specified secondary outcomes (see Table 1 and Fig. 4). Other potential covariates include participants’ relationship to the decedent, time since the death, history of exposure to other drug overdose deaths (i.e., number of people they knew who died by overdose), drug of choice (for PWUD enrolled in the RCT), and service utilization (measured using the NIH PhenX Access to Health Services Protocol derived from the National Health Interview Survey (72)).

### 2.10. Data management and analysis plan

Aim 3 will test the effect of receiving RIVER peer grief support model plus EUC compared EUC alone by contrasting outcomes of grief and using regression-based modeling to leverage all time points while adjusting for the stratification variable. We will use Intent-to-Treat analyses. Hypotheses regarding the effectiveness of the RIVER peer grief support approach will be tested with a repeated measures mixed-effects linear model, with experimental group, time (4 assessment time points), and their interaction as fixed effects. Inclusion of the interaction term will allow for examination of the difference in response trajectories between intervention arms. We will also include the stratification variable as a covariate in the model. For missing data, we will proceed with likelihood-based mixed-effects models, which are valid under the Missing at Random (MAR) assumption. For non-ignorable missingness, we will add sensitivity analyses.

### 2.11. Power

We powered our trial to detect a moderate effect size based on arm-differences of proportions of prolonged grief, due to the dearth of pilot data on mean differences. We considered an 18% arm difference in rates of prolonged grief symptoms, assuming 27% in the RIVER plus EUC arm and 45% in the EUC only arm. Since the RIVER peer grief support model has not yet been evaluated in those specifically bereaved by drug overdose deaths, we conservatively used estimates of proportions reported on rates of PGD among those bereaved by suicide (6). We will have 80% power to detect a moderate effect size of a Cohen’s d of 0.4. We based this calculation on a two-tailed test of proportions for the null hypothesis of no difference in arms, and a significance level of 0.05. We are also adequately powered to perform repeated measures analysis to detect a group by time interaction term in a mixed model to address Aim 3. With our proposed follow-up sample size (*n* = 208), we have 90% power to detect a group by time interaction, expecting a Cohen’s d of 0.4 as before, and assuming adjustment of the baseline grief score, and a correlation of 50% between repeated measures.

## 3. Discussion

Our nation is experiencing historic rates of drug overdose deaths that have left behind millions of bereaved families. People who are bereaved by overdose experience a “special grief” that compounds suffering and help-seeking. Overdose bereavement is an unfortunate and common reality for many Americans, and the ripple effect of overdose deaths may increase risk for overdose morbidity and mortality among the bereaved. No evidence-based interventions exist to support this population, and the science must catch up. Peer grief support interventions hold promise and may be especially crucial for this population given the increased stigma, isolation, and risks this population faces. This study seeks not only to understand whether a peer support model aids those grieving an overdose death, but also to explore the feasibility and acceptability of proactively engaging grievers through MEOs. In doing so, we aim to reach out to grievers who may not otherwise seek care. If our study is successful, our practice-research partnership and engagement of advisory board members will propel the translation of research to practice and address the large toll the overdose crisis takes on this overlooked population.

## Figures and Tables

**Table 1:** Outcomes evaluated in the study and the associated instruments

Proximal Outcomes		
Outcome	Operational Definition	Measured By
Connectedness	Extent to which individual feels bonded to the descendent and their environment	Continuing Bonds Scale; higher scores indicate higher degree of connectedness Multidimensional Scale of Perceived Social Support
Acceptance	Presence of maladaptive cognitions and symptoms related to complicated grief	Typical Beliefs Questionnaire; higher levels of loss acceptance are associated with deceased development of PGD
Coping	What and at what frequency coping strategies are utilized by the bereaved	The Coping Assessment for Bereavement and Loss Experiences (CABLE); higher scores indicate more frequent use of coping strategies
Grief-related Avoidance	Presence and extent of avoidance behaviors due to avoiding feelings of grief	Grief-Related Avoidance Questionnaire (GRAQ); higher scores are associated with increased occurrence of prolonged grief and increased grief symptom severity
**Distal Outcomes**		
Grief Symptoms (Primary)	Screening tool for the presence of prolonged grief	Brief Grief Questionnaire (BGQ); a score of 4 or higher is associated with PGD
**Secondary Outcomes**		
Substance Use	Items assessing the presence and frequency of substance use and substance use disorder over the past 30 days	PROMIS; a validated and reliable instrument for substance use research Tobacco, Alcohol, Prescription Medication, and Other Substances Tool (TAPS)
Depression	Instrument evaluating the severity of depressive symptoms and presence of suicidality	Beck Depression Inventory; the development of prolonged grief has been associated with increased depression severity
Trauma	Scale evaluating severity of symptoms associated with posttraumatic stress disorder	Posttraumatic Stress Disorder Checklist (PCL-5); the development of prolonged grief has been associated with higher global PCL-5 scores
Pain	Scale evaluating the intensity and interference pain experienced has on daily life and well-being	PEG; grief can cause increases in both emotional and physical pain when not addressed effectively
**RIVER Facilitator Outcomes**		
Grief	Screening tool for the presence of prolonged grief	Same measure as our Primary outcome
Posttraumatic Growth	Extent of positive outcomes experienced in individuals struggling with trauma due to addressing their trauma	Posttraumatic Growth Inventory (PTGI); intervention delivery has been associated with higher rates of posttraumatic growth than traditional therapy
Burnout	Extent of burnout experienced across one’s personal and work life	Copenhagen Burnout Inventory; toxic stress caused by burnout can interfere with the delivery of grief support services

## Data Availability

The datasets generated and/or analyzed during the current study are available in the NIH HEAL Data Repository (https://healdata.org/portal/discovery/HDP01464/)

## References

[1] Garnett MF, Miniño AM. Drug Overdose Deaths in the United States, 2003–2023. [cited 2025 Mar 13]; Available from: https://stacks.cdc.gov/view/cdc/170565

[2] SUDORS Dashboard. Fatal Overdose Data | Drug Overdose | CDC Injury Center [Internet]. 2023 [cited 2023 May 2]. Available from: https://www.cdc.gov/drugoverdose/fatal/dashboard/index.html

[3] D’Orsogna MR, Böttcher L, Chou T. Fentanyl-driven acceleration of racial, gender and geographical disparities in drug overdose deaths in the United States. PLOS Glob Public Health. 2023;3(3):e0000769. 36962959 10.1371/journal.pgph.0000769 PMC10032521

[4] Stein BD, Kilmer B, Taylor J, Vaiana ME, Barnes-Proby D, Caulkins JP America’s Opioid Ecosystem: How Leveraging System Interactions Can Help Curb Addiction, Overdose, and Other Harms [Internet]. RAND Corporation; 2023 Mar [cited 2025 Apr 22]. Available from: https://www.rand.org/pubs/research_reports/RRA604-1.html 10.7249/rhq-10-04-01 PMC10501822 37720068

[5] Lang J, Mendenhall E, Koon AD. Disentangling opioids-related overdose syndemics: a scoping review. Int J Drug Policy. 2023;119:104152. 37542742 10.1016/j.drugpo.2023.104152

[6] Powell D. Growth in Suicide Rates Among Children During the Illicit Opioid Crisis. Demography. 2023;60(6):1843–75. 38009202 10.1215/00703370-11077660 PMC12101483

[7] Levitt A, Mermin J, Jones CM, See I, Butler JC. Infectious Diseases and Injection Drug Use: Public Health Burden and Response. J Infect Dis. 2020;222(Supplement5):S213–7. 32877539 10.1093/infdis/jiaa432

[8] Rosenfeld R, Wallman J, Roth R. The Opioid Epidemic and Homicide in the United States. J Res Crime Delinquency. 2021;58(5):545–90.

[9] Stone R, Rothman EF. Opioid Use and Intimate Partner Violence: a Systematic Review. Curr Epidemiol Rep. 2019;6(2):215–30.

[10] Titlestad KB, Lindeman Sari Kaarina, Lund Hans, and, Dyregrov K. How do family members experience drug death bereavement? A systematic review of the literature. Death Stud. 2021;45(7):508–21. 31390307 10.1080/07481187.2019.1649085

[11] Athey A, Kilmer B, Cerel J. An Overlooked Emergency: More Than One in Eight US Adults Have Had Their Lives Disrupted by Drug Overdose Deaths. Am J Public Health. 2024;114(3):276–9. 38382027 10.2105/AJPH.2023.307550 PMC10882395

[12] Kennedy-Hendricks A, Ettman CK, Gollust SE, Bandara SN, Abdalla SM, Castrucci BC, Experience of Personal Loss Due to Drug Overdose Among US Adults. JAMA Health Forum. 2024;5(5):e241262. 38819798 10.1001/jamahealthforum.2024.1262 PMC11143465

[13] Verdery AM, Ryan-Claytor C, Smith-Greenaway E, Sarkar N, Livings M. More Than 1.4 Million US Children Have Lost a Family Member to Drug Overdose. Am J Public Health. 2024;114(12):1394–7. 39326002 10.2105/AJPH.2024.307847 PMC11540940

[14] Cerel J, Brown MM, Maple M, Singleton M, van de Venne J, Moore M, How Many People Are Exposed to Suicide? Not Six. Suicide Life Threat Behav. 2019;49(2):529–34. 29512876 10.1111/sltb.12450

[15] Kustanti CY, Jen HJ, Chu H, Liu D, Chen R, Lin HC, Prevalence of grief symptoms and disorders in the time of COVID-19 pandemic: A meta-analysis. Int J Ment Health Nurs. 2023;32(3):904–16. 36880520 10.1111/inm.13136

[16] Dyregrov K. Møgster, Birthe, Løseth Hilde-Margit, Lorås Lennart, and Titlestad KB. The special grief following drug related deaths. Addict Res Theory. 2020;28(5):415–24.

[17] Titlestad KB, Schmid M, Therese, Dyregrov K. Prevalence and predictors of prolonged grief symptoms among those bereaved from a drug-related death in a convenience sample of Norwegian parents: A cross-sectional study. Death Stud. 2022;46(6):1354–63. 33427100 10.1080/07481187.2020.1867255

[18] Mauro C, Tumasian RA 3rd, Skritskaya N, Gacheru M, Zisook S, Simon N, The efficacy of complicated grief therapy for DSM-5-TR prolonged grief disorder. World Psychiatry. 2022;21(2):318. 35524621 10.1002/wps.20991 PMC9077606

[19] Pizzicato LN, Johnson CC, Viner KM. Correlates of experiencing and witnessing non-fatal opioid overdoses among individuals accessing harm reduction services in Philadelphia, Pennsylvania. Subst Abuse. 2020;41(3):301–6. 10.1080/08897077.2019.1675115 31644397

[20] Frumkin MR, Robinaugh DJ, LeBlanc NJ, Ahmad Z, Bui E, Nock MK, The pain of grief: Exploring the concept of psychological pain and its relation to complicated grief, depression, and risk for suicide in bereaved adults. J Clin Psychol. 2021;77(1):254–67. 32662088 10.1002/jclp.23024 PMC7725983

[21] Bottomley JS, Feigelman WT, Rheingold AA. Exploring the mental health correlates of overdose loss. Stress Health J Int Soc Investig Stress. 2022;38(2):350–63. 10.1002/smi.3092 PMC11267624 34448352

[22] Coady A, Johnston C, Koersen B, Piercy J. Stopping the Harm: Psychosocial Outcomes of Families Affected by Drug Use. Can J Addict. 2022;13(4):44.

[23] Schneider KE, Tomko C, Nestadt DF, Silberzahn BE, White RH, Sherman SG. Conceptualizing overdose trauma: The relationships between experiencing and witnessing overdoses with PTSD symptoms among street-recruited female sex workers in Baltimore, Maryland. Int J Drug Policy. 2021;92:102859. 32712164 10.1016/j.drugpo.2020.102859 PMC7854789

[24] Bethune Scroggs L, Goodwin LR Jr., McDougal JJW. Co-Occurring Substance Use Disorders and Grief during Recovery. Subst Use Misuse. 2022;57(3):418–24. 34965842 10.1080/10826084.2021.2019771

[25] Parisi A, Sharma A, Howard MO, Blank Wilson A. The relationship between substance misuse and complicated grief: A systematic review. J Subst Abuse Treat. 2019;103:43–57. 31229191 10.1016/j.jsat.2019.05.012

[26] Afuseh E, Pike CA, Oruche UM. Individualized approach to primary prevention of substance use disorder: age-related risks. Subst Abuse Treat Prev Policy. 2020;15(1):58. 32795372 10.1186/s13011-020-00300-7 PMC7427884

[27] Selseng LB, Reime MA, Lindeman SK. Help and support for bereaved persons who use drugs: a qualitative study. Eur J Soc Work. 2023;0(0):1–13.

[28] Schlosser AV, Hoffer LD. I don’t go to funerals anymore: how people who use opioids grieve drug-related death in the US overdose epidemic. Harm Reduct J. 2022;19(1):110. 36183109 10.1186/s12954-022-00693-7 PMC9526383

[29] Christiansen SG, Reneflot A, Stene-Larsen K, Johan Hauge L. Parental mortality following the loss of a child to a drug-related death. Eur J Public Health. 2020;30(6):1098–102. 32535625 10.1093/eurpub/ckaa094

[30] Kenny KS, Kolla G, Firestone M, Bannerman M, Greig S, Flores BF, Frequency of fatal and non-fatal overdoses and response to grief and loss among people who inject drugs: An unexplored dimension of the opioid overdose crisis. Drug Alcohol Depend. 2022;237:109539. 35777172 10.1016/j.drugalcdep.2022.109539

[31] Dyregrov K, Selseng LB. Nothing to mourn, He was just a drug addict - stigma towards people bereaved by drug-related death. Addict Res Theory. 2022;30(1):5–15.

[32] ResearchGate [Internet]. [cited 2025 Apr 23]. (PDF) Understanding Grief in Social Work: Dual Process Model of Coping with Bereavement. Available from: https://www.researchgate.net/publication/381771608_Understanding_Grief_in_Social_Work_Dual_Process_Model_of_Coping_with_Bereavement

[33] Feigelman W, Bottomley, Jamison S, Titlestad KB. Examining grieving problem correlates of anticipation of the death vs. shock among overdose death and suicide bereaved adults. Death Stud. 2023;47(4):400–9. 35652152 10.1080/07481187.2022.2081267 PMC9712587

[34] Bottomley JS, Campbell KW, Titlestad KB, Feigelman W, Rheingold AA. Predictors of Stigma, Guilt, and Shame among Adults Bereaved by Fatal Overdose. OMEGA - J Death Dying. 2023;00302228231194208. 10.1177/00302228231194208 37553120

[35] Templeton L, Valentine, Christine MK Jennifer, Ford Allison, Velleman Richard, Walter Tony,. Bereavement following a fatal overdose: The experiences of adults in England and Scotland. Drugs Educ Prev Policy. 2017;24(1):58–66.

[36] Rockett IRH, Caine ED, Connery HS, D’Onofrio G, Gunnell DJ, Miller TR, Discerning suicide in drug intoxication deaths: Paucity and primacy of suicide notes and psychiatric history. PLoS ONE. 2018;13(1):e0190200. 29320540 10.1371/journal.pone.0190200 PMC5761891

[37] Death Studies. How do family members experience drug death bereavement? A systematic review of the literature | Request PDF [Internet]. [cited 2025 May 29]. Available from: https://www.researchgate.net/publication/335025377_Death_Studies_How_do_family_members_experience_drug_death_bereavement_A_systematic_review_of_the_literature 10.1080/07481187.2019.1649085 31390307

[38] Stout J. The Impact of Stigma on Family and Friends Bereaved by a Drug Overdose Death. 2022.

[39] Bottomley JS, Feigelman WT, Rheingold AA. Exploring the mental health correlates of overdose loss. Stress Health J Int Soc Investig Stress. 2022;38(2):350–63. 10.1002/smi.3092 PMC11267624 34448352

[40] Scott HR, Pitman A, Kozhuharova P, Lloyd-Evans B. A systematic review of studies describing the influence of informal social support on psychological wellbeing in people bereaved by sudden or violent causes of death. BMC Psychiatry. 2020;20(1):265. 32471407 10.1186/s12888-020-02639-4 PMC7257446

[41] Dyregrov K, Møgster B, Løseth HM, Lorås L, Titlestad KB. The special grief following drug related deaths. Addict Res Theory. 2020;28(5):415–24.

[42] Feigelman W, Feigelman B, Range LM. Grief and Healing Trajectories of Drug-Death-Bereaved Parents. OMEGA - J Death Dying. 2020;80(4):629–47. 10.1177/0030222818754669 29357755

[43] Stout JH, Fleury-Steiner B. Stigmatized Bereavement: A Qualitative Study on the Impacts of Stigma for Those Bereaved by a Drug-Related Death. OMEGA - J Death Dying. 2023;00302228231203355. 10.1177/00302228231203355 37725891

[44] Kalsås ØR, Titlestad KB, Dyregrov K, Fadnes LT. Needs for help and received help for those bereaved by a drug-related death: a cross-sectional study. Nord Stud Alcohol Drugs [Internet]. 2023 Aug 1 [cited 2025 Apr 23]; Available from: https://journals.sagepub.com/doi/full/10.1177/14550725221125378 10.1177/14550725221125378 PMC10634386 37969900

[45] Mead S, Hilton D, Curtis L. Peer support: A theoretical perspective. Psychiatr Rehabil J. 2001;25(2):134–41. 11769979 10.1037/h0095032

[46] Bartone PT, Bartone JV, Violanti JM, Gileno ZM. Peer Support Services for Bereaved Survivors: A Systematic Review. OMEGA - J Death Dying. 2019;80(1):137–66. 10.1177/0030222817728204 28871835

[47] Griffin E, O’Connell S, Ruane-McAteer E, Corcoran P, Arensman E. Psychosocial Outcomes of Individuals Attending a Suicide Bereavement Peer Support Group: A Follow-Up Study. Int J Environ Res Public Health. 2022;19(7):4076. 35409760 10.3390/ijerph19074076 PMC8998629

[48] Barker SL, Maguire N. Experts by Experience: Peer Support and its Use with the Homeless. Community Ment Health J. 2017;53(5):598–612. 28176210 10.1007/s10597-017-0102-2 PMC5438434

[49] Caparrós B, Masferrer L. Coping Strategies and Complicated Grief in a Substance Use Disorder Sample. Front Psychol. 2020;11:624065. 33536987 10.3389/fpsyg.2020.624065 PMC7848780

[50] Rowe M, Bellamy C, Baranoski M, Wieland M, O’Connell MJ, Benedict P, A peer-support, group intervention to reduce substance use and criminality among persons with severe mental illness. Psychiatr Serv Wash DC. 2007;58(7):955–61. 10.1176/ps.2007.58.7.955 17602012

[51] Lambert S, O’Callaghan Daniel, Frost N. Special death’: Living with bereavement by drug-related death in Ireland. Death Stud. 2022;46(10):2335–45. 34133259 10.1080/07481187.2021.1939461

[52] Feigelman W, Jordan JR, Gorman BS. Parental Grief after a Child’S Drug Death Compared to other Death Causes: Investigating a Greatly Neglected Bereavement Population. OMEGA - J Death Dying. 2011;63(4):291–316. 10.2190/OM.63.4.a 22010370

[53] Higgins A, Hybholt L, Meuser OA, Eustace Cook J, Downes C, Morrissey J. Scoping Review of Peer-Led Support for People Bereaved by Suicide. Int J Environ Res Public Health. 2022;19(6):3485. 35329171 10.3390/ijerph19063485 PMC8954346

[54] Whats your Grief [Internet]. [cited 2025 Apr 23]. Surviving the Grief of an Overdose Death. Available from: https://whatsyourgrief.com/product/surviving-the-grief-of-an-overdose-death/

[55] Surviving the Grief of an Overdose Death [Internet]. Whats your Grief. [cited 2025 Jun 2]. Available from: https://whatsyourgrief.com/product/surviving-the-grief-of-an-overdose-death/

[56] Thoele K, Ferren M, Moffat L, Keen A, Newhouse R. Development and use of a toolkit to facilitate implementation of an evidence-based intervention: a descriptive case study. Implement Sci Commun. 2020;1(1):86. 33043301 10.1186/s43058-020-00081-x PMC7539511

[57] Motivational Interviewing. Fourth Edition: Helping People Change and Grow [Internet]. [cited 2025 May 29]. Available from: https://www.guilford.com/books/Motivational-Interviewing/Miller-Rollnick/9781462552795?srsltid=AfmBOoralsNOgme7R7PsHTsOzEqELEvBFBxo8MaAQNy8idLX5E0reFm2

[58] miti4_2.pdf. [Internet]. [cited 2025 Apr 23]. Available from: https://motivationalinterviewing.org/sites/default/files/miti4_2.pdf

[59] Prolonged Grief Disorder [Internet]. [cited 2025 Apr 23]. Available from: https://www.psychiatry.org:443/patients-families/prolonged-grief-disorder

[60] Weiner BJ, Lewis CC, Stanick C, Powell BJ, Dorsey CN, Clary AS, Psychometric assessment of three newly developed implementation outcome measures. Implement Sci. 2017;12(1):108. 28851459 10.1186/s13012-017-0635-3 PMC5576104

[61] Attkisson CC, Greenfield TK. The Client Satisfaction Questionnaire (CSQ) Scales and the Service Satisfaction Scale-30 (SSS-30).

[62] Larsen DL, Attkisson CC, Hargreaves WA, Nguyen TD. Assessment of client/patient satisfaction: development of a general scale. Eval Program Plann. 1979;2(3):197–207. 10245370 10.1016/0149-7189(79)90094-6

[63] Blonigen DM, Harris-Olenak B, Kuhn E, Timko C, Humphreys K, Smith JS, Using Peers to Increase Veterans’ Engagement in a Smartphone Application for Unhealthy Alcohol Use: A Pilot Study of Acceptability and Utility. Psychol Addict Behav J Soc Psychol Addict Behav. 2021;35(7):829–39. 10.1037/adb0000598 PMC7769861 32597665

[64] Lewinski AA, Crowley MJ, Miller C, Bosworth HB, Jackson GL, Steinhauser K, Applied Rapid Qualitative Analysis to Develop a Contextually Appropriate Intervention and Increase the Likelihood of Uptake. Med Care. 2021;59(Suppl 3):S242–51. 33976073 10.1097/MLR.0000000000001553 PMC8132894

[65] Krippendorff K. Content Analysis: An Introduction to Its Methodology [Internet]. SAGE Publications, Inc.; 2019 [cited 2025 Apr 23]. Available from: https://methods.sagepub.com/book/mono/content-analysis-4e/toc

[66] Hennink M, Kaiser BN. Sample sizes for saturation in qualitative research: A systematic review of empirical tests. Soc Sci Med 1982. 2022;292:114523. 10.1016/j.socscimed.2021.114523 34785096

[67] O’Brien BC, Harris IB, Beckman TJ, Reed DA, Cook DA. Standards for Reporting Qualitative Research: A Synthesis of Recommendations. Acad Med. 2014;89(9):1245. 24979285 10.1097/ACM.0000000000000388

[68] Pocock SJ. Clinical Trials: A Practical Approach.

[69] 2023 NSDUH Annual National Report. | CBHSQ Data [Internet]. [cited 2025 Mar 13]. Available from: https://www.samhsa.gov/data/report/2023-nsduh-annual-national-report

[70] PhenX Toolkit. [Internet]. [cited 2025 Apr 23]. Available from: https://www.phenxtoolkit.org/

[71] HOMEPAGE | NIMHD [Internet]. [cited 2025 Apr 23]. Available from: https://www.nimhd.nih.gov/

[72] CDC. National Center for Health Statistics. 2025 [cited 2025 Apr 23]. National Center for Health Statistics. Available from: https://www.cdc.gov/nchs/index.html

